# Correlations Between Leptin Gene Polymorphisms 223 A/G, 1019 G/A, 492 G/C, 976 C/A, and Anthropometrical and Biochemical Parameters in Children With Obesity: A Prospective Case-Control Study in a Romanian Population—The Nutrichild Study

**DOI:** 10.1097/MD.0000000000003115

**Published:** 2016-03-25

**Authors:** Cristina Oana Mărginean, Claudiu Mărginean, Septimiu Voidăzan, Lorena Meliţ, Andrei Crauciuc, Carmen Duicu, Claudia Bănescu

**Affiliations:** From the Department of Pediatrics (COM, LM, CD); Department of Obstetrics and Gynecology (CM); Department of Epidemiology (SV); and Department of Genetics (AC, CB), University of Medicine and Pharmacy Tîrgu Mureş, Romania.

## Abstract

The aim of this study was to establish the manner in which the *LEPR* 223, 1019, 492, and 976 gene polymorphisms influence child obesity.

We performed a prospective case-control study on 264 hospitalized children from Romania (Nutrichild study) whom we divided into 2 groups: Group I —143 controls and Group II—121 obese children.

The 2 groups were evaluated regarding the anthropometry (MUAC, TST, H/L, hip, and abdominal circumference), paraclinical results (protein, leptin, adiponectin, TNF alfa, IL 6, IL 8, VEGF, protein, albumin) and *LEPR* 223, 1019, 492, and 976 gene polymorphisms. We noticed that the most frequent genotypes in obese children were AG+GG for *LEPR* 223 gene (*P* = 0.0001) and GA+AA for *LEPR* 1019 gene (*P* = 0.0001), whereas *LEPR* 492 and *LEPR* 976 gene polymorphisms did not correlate with obesity. MUAC, TST, H/L, leptin, and adiponectin were correlated with the GG genotype of the *LEPR* 223 gene, whereas the AG genotype correlated with TNF alpha and serum IL 8. Hip and abdominal perimeters were higher in *LEPR* 1019 AA genotype carriers, whereas TNF alpha and IL 6 correlated with the GG genotype of the same gene. Obesity did not correlate with protein serum levels.

We observed that obesity is more frequent in children with *LEPR* 223 AG+GG and *LEPR* 1019 GA+AA genotypes. In obese children *LEPR* 223/492/1019 AG/GG/GA, GG/GG/GA and AA/GG/GA combined genotypes are more frequent.

## INTRODUCTION

Obesity is determined by the combined effect of genes, environment, lifestyle, and interactions of these factors.^1^ Studies identified several critical periods when the onset of obesity is during childhood or adolescence, such as: pregnancy,^1,2^ period of childhood “adiposity rebound” development (age 3–6 years)^3^ and puberty.^4^ The increased rate of comorbidities such as dyslipidemia, hypertension, type II diabetes mellitus in overweight or obese teenagers show us that young people are not protected by metabolic disorders that come with excessive deposits of adipose tissue.^1,5,6^

In Romania, several studies with the aim of evaluating the prevalence of childhood obesity were also performed. The data resulted from these studies showed a different prevalence, depending on the geographic area. Thus, in Cluj-Napoca (Central Romania), a study from 2009 underlined a 29% prevalence of obesity and a 12.84% prevalence of overweight in school-aged children.^7^ Another study from the West of the country^8^ published in 2012 provided a prevalence of 18.2% for overweight and 7.2% for obesity; meanwhile in Craiova (Southwest), the studies performed on children aged between 3 and 14 years pointed out a 15.63% prevalence for overweight and 5.75% for obesity.^9,10^ The most alarming numbers were found in Northeastern Romania,^11^ where rates of 24% for overweight and 7% for obesity were encountered in elementary school children.

There are several mediators that intervene in appetite regulation, such as insulin, with its peripheral regulating role; gastro-intestinal peptides (cholecystokinin, peptide 1 glucagon like, peptide Y), which intervene in the appearance of satiety sensation; ghrelin, that stimulates appetite; and leptin that decreases appetite and increases energetic consumption.^12^ There are >600 genes, markers, and chromosomal regions that are involved in the development of human overweight,^12^ such as: mutations in the FTO gene (Fat mass and obesity-associated gene), MC4R, MC3R, and MC2R genes (melanocortin receptor), SIM1 (single-minded homolog) and POMC (proopiomelanocortin),^13^ SH2B1 gene (SH2B Adaptor Protein 1 gene),^14^ PPAR γ2 gene (Peroxisome proliferator-activated receptor gamma gene), and obesity gene (situated on the long arm of chromosome 7, that predisposes to obesity).^9^ In 1994 the “obesity gene” was identified in mice, which codifies leptin that owns a hormonal role, being synthetized by adipocytes. The congenital deficit of leptin leads to morbid obesity, severe hyperphagia, hyperinsulinism or type 2 diabetes mellitus, hypogonadotropic hypogonadism, hypofunction of T cells and endocrine/metabolic dysfunctions.^15,16^ Mutations in the leptin gene are associated with moderate obesity and normal function of the T cells.^17^ The serum leptin level is dependent on the age and gender, being correlated with the volume of adipose tissue.^17–19^

Childhood obesity increases the risk of adult obesity, this fact leading to the increased importance of determining the causes of childhood obesity and preventing it.^20^

One commonly studied candidate gene for obesity, the leptin receptor gene (*LEPR*), is on a biologic pathway to obesity (leptin-insulin pathway).^21^ Leptin (LEP) gene and *LEPR* gene products have defined a new biological pathway for the regulation of food intake and energy expenditure.^1^ Leptin is produced by adipose tissue and other organs and has pleiotropic actions, including regulation of several neuropeptides involved in appetite control and thermogenesis.^21^ There are studies that did not underline an association between the obesity and the leptin gene;^22,23^ meanwhile others proved the role of the leptin gene and also other factors such as gender, environmental factors, and others.^21,24^ The most frequent polymorphisms of the leptin gene involved in obesity are Q223R, K109R, and K656N.^21^ In an analysis of 38 SNPs within the *LEPR* gene (among them K109R) in a cohort of >6000 Caucasian adults from the Swiss CoLaus study,^21,25^ the authors tried to find an association with gender. In that study the rs10128072, rs3790438, and rs3790437 variants showed interaction with gender for their association with overweight, waist circumference, and fat mass in linear regressions.^21^ Although mutations in the adipokines lead to an extreme obesity syndrome, the relationship between the common single nucleotide polymorphisms (SNPs) within the genes encoding for adipokines and obesity are still controversial.^26,27^ White adipose tissue is involved in overall metabolic control, adipocytes playing an active role in extensive cross-talk with other cell types and controlling the energy metabolism within the body.^27^

The interest in LEP and *LEPR* as susceptibility genes for human obesity led to the identification of several common polymorphisms, among which *LEPR* –2548 G/A and *LEPR* Gln223Arg are of major importance.^27–30^

Cai found in 2008 that several candidate genes including the *LEPR* are involved in the development of child's obesity.^31^ Regarding polymorphisms in the leptin gene, certain studies suggested the association with obesity, including birth-weight,^32^ whereas others did not find this relationship.^33,34^

The aim of this paper was to establish the correlation between leptin gene polymorphisms and childhood obesity, to establish correlations between anthropometric parameters (BMI—body mass index, MUAC—middle upper arm circumference, and TST—tricipital skinfold thickness) and several biochemical parameters such as proteins, cholesterol, triglycerides, lipids, but also to determine correlations between the study group and the control group. Thus, we intended to determine whether *LEPR* gene polymorphisms [Gln223Arg (668A>G), Ser492Thr (1475G>C), Ala976Asp (2927C>A), Pro1019Pro (3057 G>A)] represented risk factors in the development of child obesity and to identify the polymorphism correlated with childhood obesity.

## MATERIAL AND METHOD

A case-control study was performed on 315 pediatric patients aged between 1 and 18 years, evaluated in a Pediatric Tertiary Hospital from Romania, between January 2012 and August 2015. Parents of only 278 children agreed to their children participating in our study and among them, only 264 children remained after a selection according to sex and age, in order to comply with the pairing method. The children were included in 2 groups: Group I comprised 143 controls and Group II included 121 obese children.

We included in the control group healthy children, with normal nutritional status, without any comorbidity; in the obese group patients with anthropometric parameters such as BMI, weight, MUAC, TST, hip, and abdomen circumference > + 2SD were included.^35,36^ We excluded from our study children with obesity caused by genetic factors, chronic diseases, secondary hypercholesterolemia, pediatric patients without complete clinical, laboratory and genetic evaluation as well as children whose parents did not sign the informed consent.

The 2 groups were obtained based on the inclusion and exclusion criteria mentioned above. The obese group was obtained based on the prevalence of obesity in children from Transylvania (our area). Romania has ∼20 million people; the central region of the country (which includes 6 counties Mures, Alba, Braşov, Covasna Sibiu, Harghita) has a population of 2,500,000 habitants and Mures county ∼500,000 habitants. In Mures county the subjects between 0 and 19 years old represent on average 132,000 people. According to some studies the prevalence of childhood obesity in our area is 12.84% to 29% (12.84% overweight reference and 29% obesity).^7,8,10^ Thus, the study group needed to comprise at least 115 children to be significantly statistic. To increase the comparative statistical power, the control group was bigger than the obese group.

The subjects’ parents gave written informed consent prior to inclusion in the study and the study was performed in compliance with the principles of the Helsinki Declaration, and was approved by the Ethics Committee of the University of Medicine and Pharmacy of Tîrgu Mureş (No 13/18 July 2011).

### Anthropometric Characteristics

A single trained person performed the measurements. Weight was measured with a daily calibrated scale (±10 g error), height with a pedometer evaluated in SD (0.1 cm error); MUAC was measured at the mid-point between shoulder tip and elbow, using a tape measure calibrated in centimeters, and TST was determined on the posterior area of the upper arm, using a thickness caliper (http://www.who.int/childgrowth/training/jobaid_weighing_measuring.pdf). BMI was calculated by dividing weight (kg) to standing height squared (m^2^). Abdominal circumference was measured using a tape measure at the midpoint between the rib edge and iliac crest, and reflects the abdominal disposition of adipose tissue. In order to evaluate the hip circumference, we measured the largest part of the hips, keeping the horizontal position of the tape measure. The obtained values of these parameters were converted in SDs for age and sex using the Switzerland Growth Chart-growth curves with the Growth Analyzer 3.5 version (https://www.growthanalyser.org/), the physiological reference range was between −2.0 and +2.0 SD. We considered obesity in pediatric patients with anthropometric parameters >2.0 SD.^36^

### Laboratory Investigations

All patients underwent biochemical testing for total protein and albumin, cholesterol, triglycerides, low-density lipoprotein cholesterol (LDL-chol), high-density lipoprotein cholesterol (HDL-chol), adiponectin and leptin, transaminases, glycaemia, interleukins IL 6, IL 8, tumor necrosis factor alfa (TNF-α), and vascular endothelial growth factor (VEGF).

The serum levels of total protein and albumin were measured by spectrophotometry on morning blood samples after at least an 8-hour fasting period; a Hyres 2 Sebia, 91008 Evry Cedex France microanalyzer was used. Serum proteins were considered normal at values >6.4 g/dL whereas serum albumins at >3.5 g/dL.

The levels of cholesterol, triglycerides (TG), LDL-chol, and HDL-chol were evaluated by using spectrophotometry with a Cobas Integra 400 plus automated analyzer. Cholesterol (Chol) level was considered normal at <170 mg/dL and TG level at <130 mg/dL; the percentiles for age and gender were also reported according to standard table values.^37^

TNF-α and VEGF were determined through the immune enzymatic technique ELISA sandwich type (Enzyme Linked Immuno Sorbant Assay). The labor technique respected precisely the indications of the producer's guide. Leptin and adiponectin levels were determined by ELISA using an automated immuno-diagnosis machine (leptin normal values: *F* = 7.36 ± 3.73 ng/mL, *B* = 3.84 ± 1.79 ng/mL; adiponectin normal mean values = 8880 ng/mL). IL 6 and IL 8 levels were determined by Immulite method with IL 6 and IL 8 Immulite kits (we considered the upper accepted limit in IL-6 as 10 pg/mL and 15 pg/mL for IL-8, respectively).

### Genotyping Description

A Quick-gDNA MiniPrep kit (ZymoResearch) was used for the isolation of genomic DNA. Purified DNA was extracted from whole blood using the Zymo-Spin column technology according to the instruction manual supplied with the kit. Genotyping of the *LEPR* polymorphisms [Gln223Arg (668A>G), Ser492Thr (1475G>C), Ala976Asp (2927C>A), Pro1019Pro (3057 G>A)] was carried out using the polymerase chain reaction–restriction fragment length polymorphism (PCR-RFLP) assay with previously described primers (Eurogentec, Belgium). PCR products were digested with the appropriate FastDigest enzymes from ThermoScientific (USA) as reported previously.^38–40^

### Statistical Analysis

For statistical calculations, Graph Pad 3.6 State Software, San Diego, CA, was used. To assess the normality of continuous variables (i.e., BMI, MUAC, TST, and so on), the Shapiro–Wilk test was applied. Student's *t-*test was used to assess the differences between means of continuous variables (expressed as mean ± SD), whereas differences between nonparametric variables (expressed as median, range) were compared using the Mann–Whitney test. The differences among constant variables and 3 genotype groups of the investigated polymorphism were estimated using ANOVA and Kruskal Wallis tests, an analysis appropriate for >2 groups. By using Bonferroni and Dunn's multiple comparison tests, we found the groups between whom there were statistically significant differences. To assess the associations between genotype distribution and other categorical variables, we used contingency tables and the chi-square test. We calculated the OR (odds ratio) to demonstrate the probability or susceptibility to obesity according to the given polymorphism. We interpreted all tests against a *P* = 0.05 significance threshold and statistical significance was considered for *P* values below the significance threshold.

## RESULTS

### Characteristics of the Subjects

BMI values represented the division criteria of the children. Group I (the control group) included 143 healthy control subjects. Group II comprised 121 children with BMI > 2.0 SD (obese children). Applying the Mann–Whitney test we observed significant differences between the median BMI of the 2 studied groups (*P* < 0.0001).

Mean age in the control group was 10.25 (1.0–18.0) years, whereas in the obese group it was 10.27 (1.0–17.00) years. Regarding gender distribution, we had the same distribution of girls and boys in the 2 groups (*P* = 0.29). Therefore, we can say that the groups were age- and sex-matched.

In Table 1, we showed the descriptive analysis of the anthropometric, biochemical parameters, and genotypes in the 2 groups. By applying Student's *t* test or Mann–Whitney test, we noticed a statistical difference between the group of obese children in comparison with the control group (e.g., TST, MUAC, abdominal circumference, hip, height, weight, cholesterol, glycemia, protein, albumin) (*P* = 0.0001) with the values being significantly higher in the obese group. Higher values of ALAT were observed in the obese group in comparison with the control group (*P* = 0.0001), higher values for ASAT (*P* = 0.05), and also higher values for TG (*P* = 0.005); on the other hand, no statistically significant differences were found regarding the cholesterol, HDL, LDL levels between the 2 groups.

**TABLE 1:**
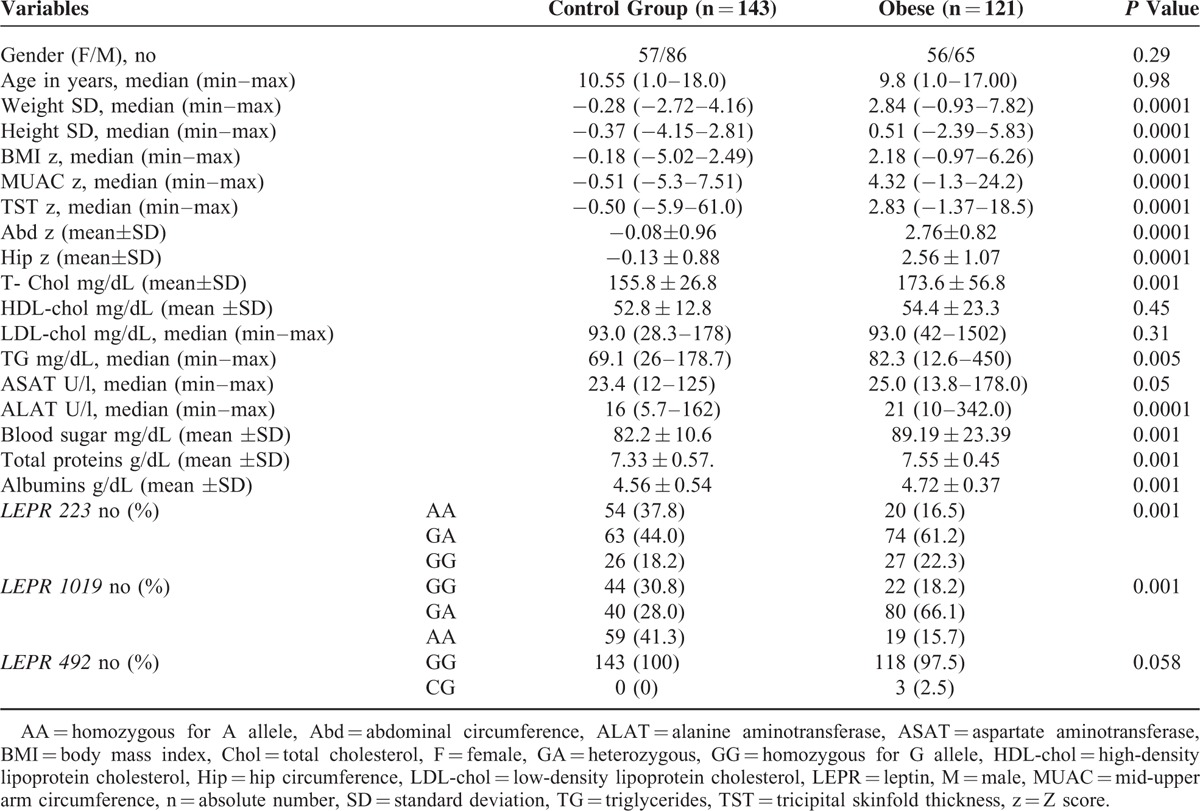
Distribution of LEPR 223, LEPR 1019, and LEPR 492 Gene Polymorphisms: Correlation With Obesity-Related Phenotypes

### LEPR Genotypes in Investigated Groups

For the 2 polymorphisms of the *LEPR* 223 and *LEPR* 1019 genes we obtained a higher incidence of the AG genotype in obese patients, and of the AA genotype in the control group (*P* = 0.001). Regarding the *LEPR* 492 polymorphism, we did not observe a statistical difference between genotypes for the 2 groups (*P* = 0.058), and for 976 *LEPR* we did not perform any statistical calculation because we identified a single AA genotype in all patients (Table 1).

### Correlation of LEPR Genotypes With Anthropometric Characteristics and Laboratory Investigations

In Table 2, we can observe that in obese children, the MUAC, TST, and leptin values associated to the *LEPR* 223 GG genotype were significantly higher than in those who present the AA genotype (*P* = 0.04, 0.03, and 0.02 respectively). For adiponectin, the values were significantly lower in obese children that present the GG genotype in comparison with the AA genotype of the *LEPR* 223 gene polymorphism (*P* = 0.032). For TNF alpha and serum IL 8, the values of those with *LEPR* 223 GA genotype were higher in comparison with those with AA genotype (*P* = 0.04 and 0.01 respectively). Also, in the obese children group in those who presented the GG genotype, the H/L values were significantly higher in comparison with the GA genotype of *LEPR* 223 polymorphism (*P* = 0.04). On the other hand, in the control group we identified significantly higher values for protein levels and MUAC in children with the AG genotype of the *LEPR* 223 polymorphism, and for leptin in those with AA genotype (*P* = 0.05/0.05/0.04) (Table 2). We have to mention that leptin values were statistically significant higher in obese comparative with the control group (*P* = 0.001), whereas those of adiponectin were smaller (*P* = 0.002).

**TABLE 2:**
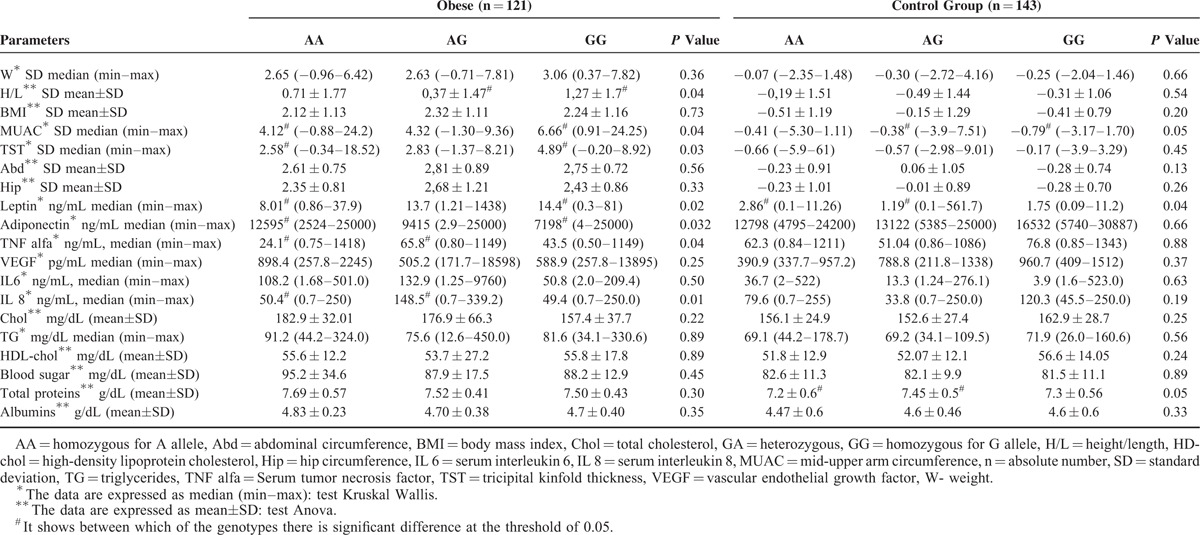
Correlations of Anthropometric and Laboratory Parameters With LEPR 223 Genotypes

In Table 3, we underline that in the obese group, the values of the abdominal and hip perimeters were higher in children with the AA variant homozygous genotype of *LEPR* 1019 gene comparative with the GG wild-type genotype (*P* = 0.05/0.04), and for TNF alpha and IL the serum values were higher in children with GG genotype in comparison with the AA genotype (*P* = 0.03/0.04).

**TABLE 3:**
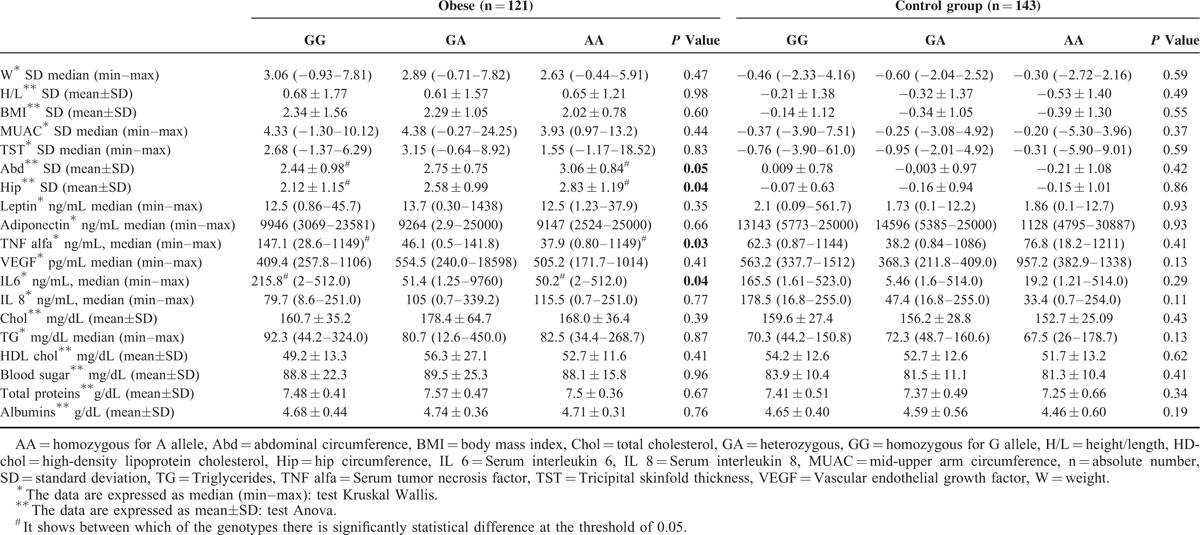
Correlations of Anthropometric and Laboratory Parameters With LEPR 1019 Genotypes

Similar to the *LEPR* 223 polymorphism, leptin values were significantly higher in obese in comparison with controls (*P* = 0.001), whereas the adiponectin levels were smaller (*P* = 0.001) for the *LEPR* 1019 polymorphism.

For 2 polymorphisms, we did not perform statistical analysis because of the small number of cases, namely 3, for *LEPR* 492, and lack in genotype variance of the *LEPR* 976 polymorphism (all the cases were variant homozygous AA).

### The Risk of LEPR 223 and LEPR 1019 Gene Polymorphisms in Development of Obesity

**FIGURE 1:**
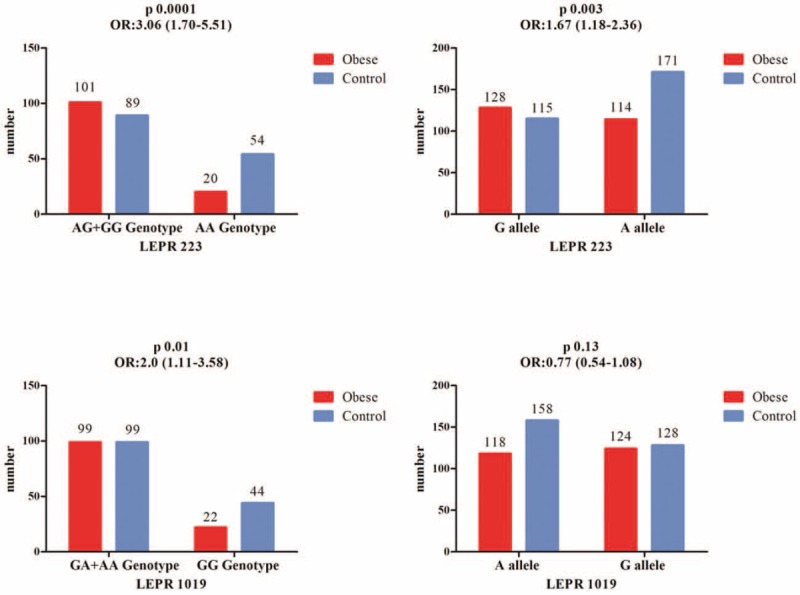
LEPR 223, LEPR 1019 gene polymorphism in obese group versus control group. ^∗^Reference group, AA = homozygous for A allele; CI = confidence interval; GA = heterozygous; GG = homozygous for G allele; n = absolute number; OR = odds ratio.

In Figure 1, considering the control group as reference and applying a bivariate analysis, we evaluated the risk for developing obesity concerning the *LEPR* 223 and *LEPR* 1019 polymorphisms, based on a contingency table.

In case of the *LEPR* 223, polymorphism comparing the combination AG+GG with the AA genotype, we observed that there was 3.06 higher risk for developing obesity in variant AG+GG genotype patients (OR 3.06; 95% CI 1.70–5.51; *P* = 0.0001) and a 1.67 higher probability for G allele carriers (OR 1.67; 95% CI 1.18–2.36; *P* = 0.0038) for developing obesity, comparative to the control group. For the *LEPR* 1019 gene polymorphism we noticed that there was 2.0 higher risk for obesity development in patients harboring the GA+AA genotype in comparison with the control group (OR 2.0; 95% CI 1.11–3.58; *P* = 0.01), but we did not find a statistical difference or a higher risk of obesity by comparing children with A allele versus children with G allele (*P* = 0.13).

### Frequency of Combined Investigated Genotypes—Comparison Between Obesity and Control Groups

Going on, we wanted to establish which of the studied polymorphism combination was more frequent in obese children and in controls, and if these combinations were correlated with the anthropometric parameters (TST and MUAC), and with the laboratory ones respectively (protein, albumins). Taking under consideration the combinations of *LEPR* 223/492/1019 gene polymorphisms, we studied only those who counted over 5 cases for every genotype combination, the rest being considered insignificant. Thus, the most frequent polymorphisms encountered in the control group were AA/GG/GG, AA/GG/GA, AA/GG/AA, GA/GG/AA, GA/GG/GA, GA/GG/GG, GG/GG/AA, and GG/GG/GA, the highest incidence being of the AA/GG/AA combination (23.1%), followed by the GA/GG/GG (22.4%), and GA/GG/AA combinations (13.3%) (the other combined genotypes being found in <10%, data not shown). In addition, we found that the AA/GG/GG combination, which represents the wild-type combined genotype, appeared more frequently in the control group (*P* = 0.0001). In the control group, for the most frequent combined genotypes, namely AA/GG/GG of the *LEPR* 223/492/1019 gene polymorphisms, we observed correlations with serum albumins (*P* = 0.04).

For the obese group, we found 8 more frequently combined genotypes for the *LEPR* 223/492/1019 gene polymorphisms, as follows: AA/GG/GG, AA/GG/GA, AA/GG/AA, AG/GG/AA, AG/GG/GA, AG/GG/GG, GG/GG/AA, and GG/GG/GA. The most frequent combination in the obese group was AG/GG/GA (41.3%), followed by AG/GG/GG (16.5%) and AA/GG/GA (12.4%). Applying statistical analysis, we obtained significant statistics only for TST (*P* = 0.04). In the obese group we noticed that the AG/GG/GA, AG/GG/GG, AA/GG/GA polymorphisms predominated, whereas in the control group other polymorphisms combinations were more frequent (AA/GG/AA, AG/GG/GG). The presence of at least 2 variant genotypes in the investigated combination (AG/GG/GA) was more frequently found in the obese group (41.3%) than in the control group (16.1%).

## DISCUSSIONS

Even though in the literature, there are several studies that tried to establish the relationship between the polymorphisms of leptin gene and obesity, few are those that identified correlations between different polymorphisms except for the *LEPR* Gln223Arg polymorphism, which was the most studied; even fewer were performed on children. Data referring to *LEPR* polymorphisms and adiposity, inflammatory status respectively are very few.

A small number of the encountered studies evaluated the relationship between Q223R, K109R, and K656N polymorphisms of the leptin gene and anthropometric parameters regarding their involvement in obesity, except for BMI,^21^ but no final data were published.

The study of Guízar-Mendoza et al,^1^ performed on 103 teenagers (55 obese and 48 thin), showed an increased incidence of the *LEPR* 223 gene variant polymorphism in patients with a higher level of insulin. Those who presented the G allele (variant genotype GG or AG) had higher heart sympathetic activity, body fat percentage, and leptin levels. Thus, they underlined an association between the *LEPR* 223 gene polymorphisms in Mexican adolescents with hemodynamic and metabolic disorders secondary to obesity, but without being associated with the BMI of these children. Similarly, in our study we observed that the leptin values associated with the *LEPR* 223 GG genotype were higher in obese children (*P* = 0.02) versus controls. Also, anthropometric parameters such as MUAC and TST and H/L IL were statistically correlated with the GG genotype. In exchange, for adiponectin, the values were indirectly proportional with the *LEPR* 223 GG, being smaller in obese children in comparison with the control group (*P* = 0.032).

The studies of Masuo et al,^24^ Mattevi et al,^41^ and Mergen et al^42^ identified an increased risk of overweight for the mutant G allele. The study of Mattevi et al^41^ reported a higher risk of overweight for the *LEPR* 223 variant G allele and waist circumference in men [OR = 2.1 (1.16–3.47)]. In our study, we did not notice any correlations between the *LEPR* 223 gene polymorphism and hip circumference nor with abdominal perimeter.

Mergen et al's study^42^ showed also o higher incidence of the G allele (223Arg = 0.380) correlated with the BMI value and independently by the genes’ loci. In contrast to these studies, in our research, by applying a multivariate regression, we obtained a 3.06 higher risk of developing obesity in the AG+GG genotypes and a 1.67 higher probability of developing obesity in children carrying the G allele of the LPER 223 gene polymorphism.

A meta-analysis performed in 2011 by Bender et al^21^—CoLaus study (Cohorte Lausannoise) on the Q223R polymorphism by ANOVAs or linear regressions, reported that 8 studies indicated the presence of variant G allele as being associated with a high risk of obesity, whereas in 5 studies a protective effect and in another 18 studies no association was found.

Even though *LEPR* 223 gene polymorphism was the most studied in adults and almost the only 1 evaluated in children, there have been no conclusions formed about it.

The studies performed on French Caucasians,^43^ Finish,^44^ and Japanese^45^ pointed out the association of *LEPR* 223 gene polymorphism with different markers of obesity and also with diabetes mellitus. *LEPR* 223 G allele was observed in obese Caucasians,^46^ and also in the Mediterranean population^47^ without any statistical difference regarding gender or age. Similarly, in our study we did not find a predisposition of the G allele toward gender or age. We did not notice significant statistical differences between the average age of the 2 groups, median age being 10.25 years in the control group and 10.27 years in thee obese group, our groups being gender and age-matched.

There are a few studies that did not find any relation between obesity and *LEPR* 223 gene polymorphisms.^33,48–50^ Among these studies, that of Constantin^48^ was performed in Romania on 202 patients (of which 108 were obese), in the same area with our study. In contrast to this study performed on adult patients, in which no correlations between the Q223R gene polymorphism and weight, height, waist circumference, waist-to-hip ratio (WHR), fat mass were observed, in our study performed on children we observed correlations with other anthropometric parameters such as MUAC, TST, and H/L which were significantly higher in *LEPR* 223 GG versus AA genotypes (*P* = 0.04/0.03/0.04). We also obtained correlations with the serum level of TNF alpha and IL 8 for the GA genotype (*P* = 0.04/0.01). Otherwise, there are studies suggesting that obesity is an inflammatory status associated with the increase of some proinflammatory cytokines such as IL 6 and IL 8, but also with the blood level of C-reactive protein.^51^

There are few studies performed on leptin gene polymorphisms in children. An Asian study performed on 136 Japanese obese children reported that *LEPR* Lys109Arg and Ser343Ser gene polymorphisms were associated with the degree of obesity, without identifying a correlation with *LEPR* 223 gene polymorphisms.^52^ Similar results reported the study of Pyrzak et al^33^ performed on 101 children, where on 60 obese patients the authors highlighted the association between *LEPR* 223 gene polymorphism and obesity. In contrast, in our study we observed a higher incidence of the *LEPR* 223 AG+GG genotypes and of the *LEPR* 1019 GA+AA in obese patients. In addition, leptin values were higher in obese children that presented the GG genotype versus AA genotype of the *LEPR* 223 gene (*P* = 0.02), varying indirectly proportional with serum adiponectin levels, which were significantly higher for the same genotype (*P* = 0.032).

The review of Bender et al^21^ did not underline an association between the combined *LEPR* Q223R, K109R, and K656N single nucleotide polymorphism and obesity-related outcomes, but for the *LEPR* Q223R polymorphism an association with overweight in studies considering a BMI cut-off value of 25 to separate normal weight from overweight was observed.

Another single nucleotide polymorphism on which we found few data, is that of the *LEPR* 1019 gene.^23^ Even though we did not find significant correlations of *LEPR* 1019 gene polymorphism in the specialty literature, in our study on obese children the values of the abdominal and hip perimeters were higher in children with AA genotype versus GG genotype of the *LEPR* 1019 gene, and for TNF alpha and IL6 the serum values were higher in children with GG genotype in comparison with AA genotype (*P* = 0.03/0.04). To our knowledge, there are not data in literature concerning the association between the *LEPR* 1019 gene polymorphism and child obesity; by applying a multivariate regression, in our study, we obtained a 2.00 higher risk of developing obesity in children for the combination GA+AA of this polymorphism.

The huge review performed by Paracchini et al^23^ in 2005 regarding the 3 polymorphisms in the *LEPR* gene (K109R, Q223R, K656N) and the 2 polymorphisms in the PPARG gene (C161T and P12A) in healthy and obese subjects suggested no evidence of an association between the genes involved in leptin regulation and obesity. The analysis stratified according to ethnicity did not show any variation across populations.^23,53^

In any case, the analysis of the *LEPR* 223 and *LEPR* 1019 polymorphisms showed that they characterize better the profile of an obese child. *LEPR* 223 polymorphism is indeed related with anthropometric (MUAC TST and I/L) and biochemical (proteins, adiponectin, leptin, TNF alfa, IL-8) parameters, whereas *LEPR* 1019 polymorphism is related with hip and abdominal circumference among the anthropometric parameters and with the serum level of IL 6 and TNF alpha. In our study, we did not obtain any statistical correlation regarding the polymorphisms of the *LEPR* 492 gene and the investigated parameters.

The most frequently encountered combinations in our study on obese patients were AG/GG/GA, AG/GG/GG and AA/GG/GA of *LEPR* 223, 492 and 1019 genes and were significantly correlated with high albumin levels (*P* = 0.04).

We must underline some limitations and minuses of our study, as follows: the relatively small number of cases included in the study—obese patients (n = 121) and normal children (n = 143), which diminished the statistical power of the study. Second, we did not evaluate the complications of obesity, especially the metabolic syndrome, nor the insulin resistance, or the manner in which the sugar, lipid, and protein intake influence the nutritional status. We had data only from a geographic region of Romania, the center of the country, on a relatively uniform sex- and age-matched Caucasian pediatric population. We did not provide data referring to the alimentary habits, environmental, geographic factors that could have an influence on the results. Due to the fact that obesity is the complex result of some genetic, environmental, and nutritional factors, we consider that it would have been useful to evaluate the gene polymorphisms also in these children’ parents or siblings, which is our future goal. In addition, we aim to extend our study on a bigger geographic area.

However, it is not meaningless the fact that it is the first study on children that studied these 4 polymorphisms of leptin, independently or in association, and which for the first time established correlations between anthropometric and laboratory parameters, including proinflammatory cytokines. Due to the fact that we did not find data in the literature regarding the *LEPR* 1019, 492 and 976 gene polymorphisms, we can consider that this study is a pilot one, and that it needs to be extended on a bigger pediatric population, and why not on an adult one.

*LEPR* 223, 1019, 492, and 976 gene polymorphisms can modulate the nutritional status in normal and also in obese children, fact that should be interpreted in the context of the multiple factors that modulate obesity such as environmental, nutritional, or social factors.

According to a multivariate analysis, the highest risk of developing obesity was owned by the children with AG+GG genotypes of the *LEPR* 223 gene, and GA + AA for the *LEPR* 1019 gene. Children carrying the G allele of the *LEPR* 223 gene also had a higher probability of becoming obese. In our study on Caucasian children from Romania, we observed that the variant genotypes GG, AG of *LEPR 223,* and GA and AA of *LEPR 1019* are the ones that appear more frequently in obese children.

## CONCLUSIONS

We can conclude that MUAC, TST, H/L, leptin, and adiponectin are correlated with the variant GG genotype of the *LEPR* 223 gene, whereas for TNF alpha and IL 8 are correlated with the AG genotype, protein serum level not being correlated with obesity, therefore being without importance in obesity determinism. For the *LEPR* 1019 gene polymorphism hip and abdominal perimeters are correlated with the variant AA genotype, whereas TNF alpha and IL 6 are correlated with the wild-type GG genotype. Our study points out the most frequent combinations of the *LEPR* 223, 492 and 1019 genes in obese children to be AG/GG/GA, AG/GG/GG and AA/GG/GA.
